# Obstructive Sleep Apnoea in Pakistan: A Single Tertiary Care Center Experience

**DOI:** 10.7759/cureus.6459

**Published:** 2019-12-24

**Authors:** Nadia Sultan, Maham Ajmal, Imad-ud-din Saqib, Amen Mobeen, Mobeen Iqbal, Farrukh Mateen, Sohail Naseem, Maimoona Siddiqui, Aamir Iftikhar

**Affiliations:** 1 Internal Medicine, Shifa International Hospital, Islamabad, PAK; 2 Dermatology, Islamic International Medical College (Riphah International University), Rawalpindi, PAK; 3 Pulmonary and Critical Care, Maroof International Hospital, Islamabad, PAK; 4 Neurology, Shifa International Hospital, Islamabad, PAK; 5 Pulmonology, Shifa International Hospital, Islamabad, PAK; 6 Pulmonology/Sleep Medicine, Shifa International Hospital, Islamabad, PAK

**Keywords:** sleep, sleep medicine, sleep disorders, obstructive sleep apnea, polysomnography

## Abstract

Introduction: Considerable interest has been shown in the field of sleep medicine in recent decades. Obstructive sleep apnoea (OSA) is a common condition that remains neglected in most parts of the world. Data are scarce, if any, when it comes to developing countries. We sought to describe the patient population in a single private tertiary care center from such a country.

Materials and Methods: A cross-sectional study that included a total of 203 patients over a five-year period was conducted. Polysomnographic studies were conducted in a dedicated sleep laboratory, under the supervision of sleep physicians. Data were described and analyzed based on clinical and self-reported outcomes, as well as polysomnographic characteristics, and compared them between genders and severity.

Results: With the participants having an average age of 50.84 years and a BMI of 34.7 kg/m^2^, the study found that the increase in age and BMI was significantly correlated with an increase in the severity of obstructive sleep apnea in the Pakistani population. There was a significant difference in sleep latency (20.6 min in women vs. 10.8 min in men; p-value = 0.001) and efficiency (63.7% in women vs. 69.8 in men; p-value = 0.02) between the two genders. Decreases in nadir saturation, total sleep time, and sleep latency were also associated with an increase in the level of severity.

Conclusion: There is a dire need for Pakistani, and in extension Asian, medical professionals to ramp up their pace to meet the needs of their population with regard to sleep medicine.

## Introduction

With the increasing improvements in healthcare and the general standard of living, interest is increasing in conditions that previously had little or no value. Many of these conditions indirectly affect mortality rates by helping in the management of related diseases. Sleep-disordered breathing (SDB) is one of such conditions, whose importance has just been recently recognized [1]. Awareness among physicians, let alone the general population, remains low despite its high prevalence in the general population. When left untreated, SDB has resulted in cardiovascular disease, obesity, diabetes, mental health disorders, reduced work productivity, road traffic accidents, and even death [2-6].

Obstructive sleep apnoea (OSA) comprises a major part of the spectrum of SDB. It is characterized by recurrent episodes of complete or partial obstruction of the upper airway during sleep, resulting in breathing pauses (apnoeas or hypopneas), snoring and frequent awakenings, leading to sleep fragmentation, and eventually excessive daytime sleepiness due to an unrefreshed sleep [7]. At the physiological level, there is insufficient oxygenation and release of stress hormones, which then result in the aforementioned adverse health conditions.

Most of the available data on the prevalence and risk factors of OSA come from studies conducted among whites of European descent, US Hispanics, and African American populations [8]. These studies have suggested and shown differences among racial groups that imply a role of cultural and environmental factors. Despite the lack of data in Asia, the prevalence is reported to be around 2.1%-7.5% [9-10]. In Pakistan, studies conducted in Karachi, based on the Berlin questionnaire, revealed that 10%-12.4% of the population has a high risk of OSA [11-12]. Another study conducted in Lahore was intended to describe the characteristics of a small population of 30 patients diagnosed with this condition by formal overnight polysomnography (PSG) [13].

This study attempts to evaluate a large group of Pakistani population diagnosed with OSA, describing its demographic, clinical and polysomnographic characteristics, including its associated conditions and risk factors. The study sought to examine the effects of OSA on sleep architecture, the association between OSA and body mass index (BMI), gender influence, and the correlation between severity to clinical and polysomnographic parameters in our population.

## Materials and methods

Study design

The study was a cross-sectional observational study, conducted at Shifa International Hospital after obtaining approval from the Hospital ethics committee and approval from the Institutional Review Board (reference no: 946-221-2018).

Setting

Individuals who underwent an overnight PSG study from the period of January 2013 to May 2018 at Shifa sleep disorder centre, Islamabad, were included. This is a two-bed sleep laboratory run by a multidisciplinary team of sleep physicians, neurologists, and neurophysiologists. These patients were mainly characterized by symptoms of snoring and/or excessive daytime somnolence, which were advised of this research or presented with some other conditions, but were found to have symptoms suggestive of SDB and eventually referred to the study.

Participants

All patients who underwent a PSG study within the aforementioned period of time were included. Informed consent was obtained and the procedure was explained before proceeding with the test. Patients completed the questionnaires under the supervision of a well-trained personnel in accordance with the hospital’s policy. Patients with incomplete medical records and patients who underwent a second PSG test were excluded from the study.

Operational definitions

a) OSA is defined by the presence of at least five obstructive respiratory events (apnoeas, hypopnoea, or respiratory effort-related arousals) per hour of sleep during an overnight PSG study. OSA was considered mild when apnoea hypopnoea index (AHI) ≥ 5 and < 15, moderate when AHI ≥ 15 and ≤ 30, and severe when AHI > 30.

b) AHI refers to the number of apnoea plus hypopnoea events per hour of sleep.

c) Apnoea was considered to be greater than 90% reduction in airflow that lasted for at least 10 s from baseline, while hypopnoea reduced ventilation by at least 50%, resulting in a 4% or more decrease in saturation due to partial airway obstruction or event-related arousal.

d) The Sleep Architecture described the various stages of sleep experienced by the participant.

e) Sleep Efficiency was determined by taking the ratio of the total amount of time slept to the total amount of time spent in bed.

f) Continuous Positive Airway Pressure (CPAP) is a device that is titrated according to the Academy of Sleep Medicine (AASM) manual guidelines, which allows the technologist to increase the positive airway pressure throughout the polysomnographic recording in order to determine the single fixed pressure that would eliminate respiratory disturbances.

g) Hypertension is defined as participants on any antihypertensive medication with a physician’s prescription of any specialty at the time of study or those who began treatment during the course of the study.

h) BMI was calculated by dividing the body weight (kg) by the square of the height (m). A BMI range of 25-29.9 was considered overweight, and 30 and above as obese.

i) Craniofacial abnormalities comprised a history of nasal obstruction or any other facial anatomical deformity affecting breathing.

j) Cardiovascular Complications encompassed a history of chest pain or discomfort diagnosed as angina by a cardiologist, a history of arrhythmias/heart block or myocardial infarction, or a history of coronary artery stenting, stroke, or transient ischemic attack (TIA) perceived based on information provided by patients through questionnaires.

k) Mallampati Score was assessed on the spot by a physician, asking the patient to open his mouth as wide as possible with a full tongue protrusion and no sounds. A standard I to IV grading system was used based on the visibility of the soft palate, the uvula, and the base of the tongue; class I: soft palate, fauces, uvula and visible pillars, class II: soft palate, fauces and visible uvula, class III: soft palate and visible uvula base, and class IV: soft palate not visible at all.

l) Epworth Sleepiness Scale (ESS) > 10 was considered significant for excessive daytime somnolence [14].

m) Berlin Questionnaire : the risk was labeled significant when symptoms mentioned in Sections 1 and 2 were persistent (>3-4 times/week) from the symptoms listed. Section 3 was positive when there was a history of hypertension or BMI ≥ 30 kg/m2. According to this questionnaire, to be considered as a high risk of sleep apnoea, an individual had to be positive in at least two of the three abovementioned sections [15].

Data sources and measurement

We sorted baseline data based on the subject’s records, such as age, gender, BMI, and comorbid conditions. The self-reported questionnaire based on the Berlin questionnaire, the Mallampati score, and the ESS filled out by subjects before the precedence of the study were also analyzed, while polysomnographic data were retrieved from computer-based software. A standard polygraph, utilizing polysmith software, was used to record the electroencephalogram (EEG), electrocardiogram (ECG), electrooculogram (EOG), electromyogram (EMG) of the chin and bilateral tibialis anterior muscles, airflow measurements, and chest wall and abdominal movements. Airflow was measured using nasal and oral thermistors, and a nasal pressure transducer. Respiratory effort was monitored with piezoelectric belts to measure the movement of the chest and abdomen. Oximetry was measured using a finger probe placed on the index finger. The study used snore microphones to record snoring; these were attached to the neck along with other audio-visual recording devices. All studies were analyzed by trained technicians and sleep physicians using the criteria outlined by the AASM and in concordance with further scoring updates provided by AASM.

Data entry and statistical analysis

Data entry and analysis were performed using SPSS version 24. After ensuring correct data entry, patient identifiers were removed. Descriptive statistics were used to report age, gender, BMI, symptom presentation, self-reported questionnaire scores (Mallampati, Berlin, and ESS), AHI, and sleep architecture. Subsequently, multivariate analysis was used for the same parameters while controlling other parameters. The p-value of less than 0.05 was considered significant.

## Results

A total of 300 patients with suspected OSA underwent a comprehensive diagnostic PSG in the specified period of time. Of these, 97 patients were excluded on the basis of incomplete records, leaving 203 patients eligible to qualify for the study. There were 134 (66%) men and 69 (34%) women with an average age of 50.84 years and a mean BMI of 34.7 kg/m2.

Clinical and self-reported questionnaire

Loud snoring was invariably present in a majority of the patients (63%). Daytime sleepiness was the second most commonly reported symptom (25%), and the mean ESS score was 13.7. The majority of patients (64.6%) were positive in all three categories of the Berlin questionnaire, with 32.2% being positive in two categories.

Three-quarters of the patients (74%) complained of other symptoms as well, in association with or without the two most common symptoms mentioned earlier. These included morning headaches and fatigue. The history of addiction is mainly related to smoking (9.6%). OSA severity was correlated with baseline characteristics and found a robust relationship between OSA severity and BMI; BMI >30 was itself an independent predictor of OSA severity (p-value = 0.05). Age, in general, appears to have a statistically significant effect on OSA severity (p-value < 0.000). Table 1 describes baseline patient characteristics, including risk factors.

**Table 1:** Baseline characteristics of patients along with risk factors. BMI = body mass index, OSA = obstructive sleep apnoea, LS = loud snoring, EDS = excessive daytime sleepiness, DM = diabetes mellitus, HTN = hypertension, *percentage of subjects reported on self-reported questionnaire

Baseline characteristics	Mean	Range	Standard deviation	
Age	50.8	13-90	±13.4	
BMI	34.7	18.5-50	±7.0	
Epworth sleepiness score	12.5	0-24	±6.07	
Witnessed apnoeas*	71.3%	Not applicable		Not applicable
Symptoms other than LS and EDS*	74%	Not applicable		Not applicable
Nasal obstruction*	37%	Not applicable		Not applicable
Smoking*	7%	Not applicable		Not applicable
DM*	28%	Not applicable		Not applicable
HTN*	56%	Not applicable		Not Applicable
Cardiovascular complications*	18.2%	Not applicable		Not applicable
Other diseases present*	80.0%	Not applicable		Not applicable

Polysomnographic characteristics

With regard to OSA severity, 14.2% of the patients had mild severity, 14.7% had moderate, while 50.7% had severe OSA and 17.7% of patients did not even qualify as mild OSA. The mean CPAP requirement was 11.7 cm of water. Table 2 describes the PSG characteristics.

**Table 2:** Baseline PSG characteristics of patients. PSG = polysomnography, min = minutes, AHI = apnoea hypopnoea index, NREM = non-rapid eye movement, TST = total sleep time, REM = rapid eye movement, *includes split night and diagnostic studies

PSG characteristics	Mean	Standard deviation	Range
Total sleep time (min)	173	±115	(5-695)*
Sleep latency	14.0	±19	(0.06-147)
Sleep efficiency (%)	67.4	±18	(5-97)
Nadir saturation	75.3	±14	(50-97)
AHI	41.0	±37	(0-167)
NREM 1 (% of TST)	21	±18	(0-100)
NREM 2 (% of TST)	64.0	±20	(0-210)
NREM 3 (% of TST)	3.7	±7	(0-32)
REM (% of TST)	10.3	±10.6	(0-53)

Comparison by gender

Women undergoing PSG were, on average, older compared to men and had a higher BMI. They were also more likely to suffer from comorbid conditions (diabetes mellitus, hypertension) and were more likely to have complaints of daytime somnolence and witnessed apnoea.

We did not notice any significant difference with regards to the three scoring methods (Mallampati, Berlin questionnaire scores, and ESS) between the two genders. However, during PSG, significant differences were observed for sleep latency and sleep efficiency. Table 3 details the data comparisons between the two genders, along with significant and nonsignificant differences.

**Table 3:** Data comparison and analysis according to gender. BMI: body mass index, DM: diabetes mellitus, HTN: hypertension, N: total number, min: minutes, %: percentage, NREM: non-rapid eye movement, REM: rapid eye movement. *between moderate and severe group

	Statistics	Females	Males	p value
Severity:				
Mild (AHI=5-15) Moderate(AHI=15-30) Severe(AHI=>30)	Number	8(11%) 13(18%) 33(47%)	21(15%) 17(12%) 75(55%)	0.93*
BMI	Mean	37.9	33.1	<0.000
Age (years)	Mean	55.9	48.2	<0.000
Presence of DM	Percentage	39% (N=27)	22.7% (N=30)	<0.000
Presence of HTN	Percentage	73% (N=51)	47% (N=67)	<0.000
Presence of nasal obstruction	Percentage	29.4% (N=20)	41.3% (N=55)	0.057
Daytime somnolence	Percentage	91.4 (N=63)	78% (N=104)	0.014
Witnessed apnoea	Percentage	82% (N=57)	65.7% (N=87)	0.030
Epworth sleepiness score	Mean	13.5	13.9	0.76
Berlin questionnaire one category two categories three categories	Percentage +ve	3% (N=2) 19.4% (N=13) 74.5% (N=50)	1.5% (N=2) 34.8 (N=45) 61.2% (N=80)	0.147

Comparison by severity

Although gender and the presence of comorbid conditions had no effect in determining the severity of OSA, there was a significant concordant increase in severity with increasing BMI (p-value <0.000). Patients with increased severity were more likely to have advanced age, daytime somnolence, higher Berlin questionnaire, Mallampati, and ESS. In addition, during PSG, decreases in nadir saturation, total sleep time and sleep latency were associated with an increase in the level of severity. Table 4 compares data based on different levels of severity.

**Table 4:** Data comparison and analysis according to severity. BMI = body mass index, DM = diabetes mellitus, HTN = hypertension, N = total number, min = minutes, % = percentage, NREM = non-rapid eye movement, REM = rapid eye movement

	Statistics	Normal N=36	Mild N=29	Moderate N=30	Severe N=108	p value
Gender						
Male Female	Number	21 15	21 8	17 13	75 33	0.36
Age (years)	Mean	44	52.1	52.7	52.1	0.017
BMI	Mean	31.3	31.7	35.9	36.3	<0.000
Presence of DM	Percentage +ve	3%	2%	5.9%	16%	0.12
Presence of HTN	Percentage +ve	6%	8.3%	8%	32%	0.20
Presence of nasal obstruction	Percentage +ve	4%	5.4%	4.9%	22.2	0.29
Daytime somnolence	Mean %	69	72.4	86.6	88.7	0.028
Witnessed apnoea	Percentage +ve	58	68.9	70	76.6	0.27
Epworth sleepiness score	Mean	8.5	9.5	10.7	15.0	<0.000
Berlin questionnaire one category two categories three categories	Percentage +ve	1.5(N=3) 20%(N=12) 12%(N=16)	100(N=1) 18%(N=11) 11%(N=15)	0(N=0) 15%(N=9) 15%(N=20)	0(N=0) 44%(N=26) 60%(N=79)	0.009
Malampati score 1 2 3 4	Percentage +ve	9 12 8.1 12	13 18 10 2.5	21 24 10 7.6	26 45 70 76.9	0.002
Total sleep time (min)	Mean	300	175	158	136	<0.000
Sleep latency (min)	Mean	26.8	12	11.2	11.3	<0.000
Sleep efficiency	Mean %	73	72.1	65.3	65.9	0.092
Nadir saturation	Mean %	83	80.0	77.9	70.0	<0.000
NREM stage 1	Mean %	14.5	18.2	22.5	23.5	0.055

## Discussion

We noted that a middle-aged overweight man is a typical OSA patient in our population. A woman with OSA is usually of an older age and BMI, suffers from other comorbidities, and is more aware of her symptoms before presentation. Severity in both genders increases with increasing BMI and age, a greater report of the symptoms, greater scores in the questionnaires, and a decrease in nadir saturation during sleep study.

Previously, extensive research has been done with regard to the characteristics of multiple populations in relation to OSA, most of which are whites of European descent, US Hispanics and African Americans, and to a limited extent among other populations [7, 10, 13]. A general male predominance has been observed in some studies, ranging from less than 2.3:1 to 7:1 [1]. Our data shows a similar ratio of 2:1.

Young et al. described that those with breathing pauses were three or four times more likely to have AHI >15 [1]. However, our study does not support any such link that associate a witnessed apnoea with OSA severity. The median age of women was 55.9 years, indicating that postmenopausal status in our population may suggest a possible role for menopause in SDB.

Studies describe a direct relationship between age and BMI with OSA severity, and an overall greater BMI observed among women [16]. The study also noticed an increase in severity with increasing BMI, along with the aforementioned gender differences. Our study showed a significant relationship between OSA severity and age.

Samson et al. and Tasali et al. demonstrated the presence of comorbid conditions (hypertension, type 2 diabetes mellitus, congestive heart failure, and metabolic syndrome) to be positively correlated with OSA severity [17-18]. This study failed to illustrate any of such relationship in our population. However, we noted that women diagnosed with OSA in our population were more likely to have comorbid conditions, possibly due to higher ranges of BMI reported alongside. However, our study highlighted another finding, which states that subjects with severe OSA have a four-fold increased risk of having hypertension when compared with moderate levels (32% vs. 8%). This is consistent with the views of Nieto et al. suggesting that there is a greater probability of arterial hypertension with an increased severity of OSA [19].

It is postulated that the Asian population has distinct craniofacial features based on cephalometric data that predispose them to SDB in a relatively low range of BMI in OSA patients [20]. A third of our population falls into this category in the form of nasal obstruction.

Witnessed apnoeas and daytime somnolence were more frequently reported by women, a finding that has also been observed previously [21]. A survey carried out in the last decade indicated that men and women respond to sleepiness questions differently [22]. Other studies, including our study, question the validity of such claims with results demonstrating that men and women perceive differently, thereby responding differently to the subjective sleep outcomes [22]. This difference could be eliminated using objective measures. However, in our study, both subjective and objective measures yielded similar results.

Tan et al. only recently in 2017, pointed out that the Berlin questionnaire may be useful as a screening tool in the general population due to its high sensitivity (76.9%) and its negative predictive value (96.3%) [23]. The study data show a significant correlation of OSA severity with the Berlin questionnaire, Mallampati score, and ESS. Unfortunately, due to lack of a control group, we were unable to determine its use as a screening tool for our population.

O'Connor et al. observed that the rapid eye movement (REM) difference was greater in women than in men with OSA of all degrees of severity [24]. The current study failed to depict any REM duration difference between the two genders.

Some studies suggest that the extent of nocturnal hypoxemia is a direct determinant of OSA severity [25]. Our study confirms this link, which can also further influence daytime symptoms and play a role in increasing blood pressure and the risk of cerebrovascular and cardiovascular events.

Krishnan and Collop noted better sleep efficiency in women compared to men for the same OSA severity, which is also supported by our study [26]. This may be explained by neurochemical mechanisms regulating sleep and wake patterns that are sensitive to the effects of estradiol [27]. However, the exact role of estrogen in modulating sleep patterns in the brain remains unknown.

Kim et al. advocated that short sleep duration (< 5 h) has a higher likelihood of increased AHI and visceral obesity [28]. This finding is also reflected in our study, as more time taken to sleep was linked to increased OSA severity; greater time taken to sleep implies shorter sleep duration.

Strengths and limitations

Even though our study includes the largest number of patients to date of this particular population, the sample size is still a major limitation, rendering many analyses difficult and many difficult-to-explore relationships. An example of this is the deleterious effects of SDB in the cardiovascular state. Only 18% of our patients had cardiovascular complications in the form of congestive heart failure (CHF), ischemic heart disease (IHD), or stroke; hence it was difficult to establish any statistically significant relationship.

All patient populations included were from a private tertiary care hospital. In a developing country like ours, the majority of the population is faced with affordability issues undergoing lifesaving procedures in such environments, let alone those that improve the quality of life. However, until the moment public hospitals can cater to such facilities, the brunt of such diseases is left for the private sector to bear and therefore, private sector patients constitute the bulk of diagnosed OSA patients.

With the return of foreign-trained medical specialists in low- and middle-income countries, medical facilities are being developed further. At the same time, the lack of sufficient assisting personnel results in disparity between the services provided by the same individual in his trained country and in his country of origin. This may also be due to increased burden on patient in that particular specialty or immaturity on the part of organizations or individuals overseeing quality control or a combination of both. This is evident from the observation that we excluded almost one-third of the total patients in the selected period of time due to incomplete records. The low quality of data is also a reason for the use of relatively vague criteria to describe comorbid conditions in our studies rather than the standard diagnostic criteria.

## Conclusions

In short, this study describes epidemiology of OSA through polysomnographic data. OSA is a prevalent disorder in Pakistani population causing significant morbidity in both genders. Sleep-related disorders should be taught at all levels of medical education. The increased awareness in public and general practitioners can help in early recognition and timely referral to sleep specialists. We hope that this study will highlight the importance of further research in sleep-related disorders in the Pakistani population. 
